# Induction of heparanase by HPV E6 oncogene in head and neck squamous cell carcinoma

**DOI:** 10.1111/jcmm.12179

**Published:** 2013-11-28

**Authors:** Nir Hirshoren, Raanan Bulvik, Tzahi Neuman, Ariel M Rubinstein, Amichay Meirovitz, Michael Elkin

**Affiliations:** a Department of Otolaryngology, Head & Neck Surgery, Hadassah-Hebrew University Medical CenterJerusalem, Israel; b Sharett Institute of Oncology, Hadassah-Hebrew University Medical CenterJerusalem, Israel; c Department of Pathology, Hadassah-Hebrew University Medical CenterJerusalem, Israel

**Keywords:** heparanase, head and neck cancer, human papillomavirus

## Abstract

High-risk human papillomavirus (HPV)-positive head and neck squamous cell carcinomas (HNSCCs) are highly invasive; however the identity of downstream effectors responsible for their aggressive phenotype remains underinvestigated. Here, we report that HPV-mediated up-regulation of heparanase enzyme can provide mechanistic explanation for augmented invasiveness of HPV-positive HNSCCs. Heparanase is the sole mammalian enzyme (endo-β-d-glucuronidase) degrading heparan sulphate glycosaminoglycan, key polysaccharide of the extracellular matrix. Cleavage of heparan sulphate by heparanase leads to disassembly of extracellular barriers, enabling local invasion and metastatic spread of the tumour, and releases heparan sulphate-bound growth factors from the extracellular depots. Heparanase is tightly implicated in head and neck cancer progression; yet, molecular mechanisms underlying transcriptional activation of the heparanase gene in HNSCC are largely unknown. We found that HPV16 oncogene E6 is capable of inducing overexpression of heparanase in HNSCC. Notably, radiation treatment dose-dependently suppresses E6-induced heparanase expression *in vitro*. Our results provide the first evidence for a functional involvement of HPV in heparanase induction in head and neck tumourigenesis and, given ongoing clinical testing of several heparanase-inhibiting compounds, offer important avenue for future therapeutic exploration in HNSCC, as well as other HPV-associated malignancies (*i.e*. cervical carcinoma).

## Introduction

High-risk human papillomavirus (HPV) infection is an important mechanism underlying development of head and neck squamous cell carcinomas (HNSCCs). The incidence of HPV-positive oropharyngeal squamous cell carcinoma has been steadily increasing during the last decade and currently is almost equivalent to HPV-negative oropharyngeal tumours 1,2. HPV16 is the type linked with the great majority of HPV-positive HNSCC. HPV16 viral oncogenes E6 and E7, largely responsible for HNSCC tumourigenesis, are best known for their ability to target the tumour suppressors p53 and pRb, respectively 1. Human papillomavirus-positive HNSCC tumours are characterized by high expression of p16, as a result of pRb inhibition by E7, which binds the cullin2 ubiqitin ligase complex and silences pRb. Similarly, E6 associates with E3 ubiqitin ligase (E6AP), resulting in inappropriate targeting of p53 for proteasomal degradation (reviewed in 1,2). Human papillomavirus-positive tumours form a distinct group within HNSCC, characterized by more aggressive phenotype (HPV positivity significantly correlates with both lymph node metastasis and tumour depth of invasion 3), but at the same time associated with a more favourable treatment response 1,2,4. Despite the wealth of data describing primary molecular mechanisms of HPV-mediated tumourigenesis, the identity of downstream effectors responsible for the distinct biological and clinical behaviour of HPV-positive HNSCC remains underinvestigated.

Heparanase is a single mammalian endoglycosidase capable of degrading heparan sulphate (HS), the main polysaccharide component of the extracellular matrix (ECM) 5–7. Heparanase is well-recognized as an important effector in cancer progression, neovascularization and aggressive behaviour 7–12, acting through breakdown of extracellular barriers for cell invasion and release of HS–bound angiogenic and growth factors (*i.e*. bFGF, VEGF, HGF) from the ECM depots 8,9,13–17. Direct evidence for a crucial role of the enzyme in tumour progression was provided by demonstration of enhanced aggressiveness of numerous cancer cell types following overexpression of heparanase 8,18,19, as well as inhibition of the tumourigenic/metastatic abilities of cancer cells following heparanase silencing 10,18,20–22. Causal involvement of heparanase in oral cancer is particularly well-documented 23–27. While normal oral epithelium is negative for heparanase, overexpression of the enzyme is a characteristic feature of HNSCC. Elevated levels of heparanase were also detected in the saliva of oral cancer patients 24. Heparanase up-regulation correlates with the invasiveness of oral cancer cell lines 23,26,27 and with oral tumour aggressiveness 24,26–28, resembling clinical/biological characteristics of HPV-positive tumours 1,3.

This resemblance, taken together with the established role of both HPV and heparanase in HNSCC tumourigenesis, prompted us to examine the effect of HPV16 oncogenes on heparanase expression in head and neck cancer.

## Materials and methods

### Cell culture, plasmids and transfection

CAL-27 and SCC-25 human oral squamous carcinoma cells were a generous gift from Dr. I. Vlodavsky, Technion, Haifa, Israel. CAL-27 cell line was isolated from the tissue taken prior to treatment from a 56-year-old male (site of origin: middle of the tongue; doubling time: 35 hrs) 29. SCC-25 line was isolated from a 70-year-old male (site of origin: oral cavity; TNM stage T2N1, doubling time: 35 hrs) 30. Both lines are HPV-negative, extensively characterized and widely used in oral squamous cell carcinoma (OSCC) *in vitro* studies and xenograft models 31. PCR analysis with primers specific for HPV16 E6 and E7 confirmed that SCC25 and CAL27 cells did not harbour E6 or E7 DNA. Cells were transfected using the JetPrime Transfection Kit (Polyplus-transfection SA, Illkirch, France) with either pLXSN expression vector encoding the HPV16 E6 and E7, pEGFP-C3 vector encoding for E6, or pJS-55 vector encoding for E7, or with the corresponding control empty (Vo) vector. To obtain stably transfected cells, in some experiments cells were selected with 500 μg/ml G418 (Sigma-Aldrich, Rehovot, Israel). To rule out the possibility of insertional mutagenesis, all the experiments involving stably transfected cells have been conducted using a pooled population of clones, each containing over 100 clones mixed together.

### Analysis of heparanase gene expression by quantitative real-time PCR (qRT-PCR)

Total RNA was isolated from the cells using TRIzol (Invitrogen, Carlsbad, CA, USA), according to the manufacturer's instructions, and quantified by spectrophotometry. After oligo (dT)-primed reverse transcription of 500 ng total RNA, the resulting single-stranded cDNA was amplified using real-time quantitative PCR analysis with an automated rotor gene system RG-3000A (Corbett research, Sidney, Australia). The PCR reaction mix (20 μl) was composed of 5 μl QPCR sybr master mix (Finnzymes, Vantaa, Finland), 5 μl of diluted cDNA (each sample in a six-plicate) and a final concentration of 0.3 μM of each primer. PCR conditions were as follows: an initial denaturation step at 95°C for 10 min.; 40 cycles of denaturation at 94°C for 15 sec., hybridization at 58°C for 30 sec. and elongation at 72°C for 30 sec. Actin primers were used as an internal standard. The following primers were used: E6 – S: 5′-GCTAGCATGCACCAAAAGAGAACTGC-3′, AS: 5′-TCTAGATTACAGCTGGGTTTC-3′; E7 – S: 5′-CAGAGGAGGAGGATGAAATAGA-3′, AS: 5′-CGAATGTCTACGTGTGTGCTTT-3′; β-Actin – S: 5′-TCCCTGGAGAAGAGCTACG-3′, AS: 5′-GTAGTTTCGTGGATGCCACA-3′; Heparanase – S: 5′-CTGATGTGGAGGAGAAGTTTACG-3′, AS: 5′- GTTATACCCCTTGGAAGAGCA-3′.

### Antibodies

Immunoblot analysis and immunostaining were carried out with anti-heparanase monoclonal antibody 01385-126 32,33, kindly provided by Dr. P. Kussie (ImClone Systems Inc., New York, NY, USA) and anti-p16 (R&D Systems, Minneapolis, MN, USA).

### Immunoblotting

Equal protein aliquots (60 μg) were subjected to SDS-PAGE (10% acrylamide) under reducing conditions. Proteins were transferred to a polyvinylidene difluoride membrane (Millipore Corporation, Billerica, MA, USA) and probed with the anti-heparanase monoclonal antibody 01385-126 32,33, kindly provided by Dr. P. Kussie (ImClone Systems Inc.; 1:1000), followed by horseradish peroxidase-conjugated secondary antibody (KPL) and a chemiluminescent substrate (iNtron Biotechnology, Gyeonggi-do, South Korea). Membrane was stripped and incubated with anti-β-actin (1:1000) antibody to ensure equal protein load.

### Immunohistochemistry

Formalin-fixed, oral squamous carcinoma tissues from 23 non-selected patients (average age: 59 years; 12 men and 11 women, without epidemiological differences between two groups) were available from the Dept. of Pathology, Hadassah Medical Center, Jerusalem. Comparing TNM classification, average tumour staging in p16-positive patients was T3, while in p16-negative patients average staging was T2. There were no differences in lymph node involvement and metastatic spread. The use of these specimens and data in research were approved by the Ethics Committee of the Hadassah Medical Center. Paraffin-embedded slides were deparaffinized and incubated in 3% H_2_O_2_. Antigen unmasking was carried out by heating (20 min.) in a microwave oven in 10 mM Tris buffer containing 1 mM EDTA. Slides were incubated with primary antibodies diluted in CAS-Block (Invitrogen) or with CAS-Block alone, as a control. Appropriate secondary antibodies were then added and slides incubated at room temperature for 30 min. Controls without addition of primary antibody showed low or no background staining in all cases. Human papillomavirus status of the tumour specimens was determined by the presence of diffuse nuclear and cytoplasmic staining pattern of p16, which is regarded as a suitable surrogate marker for HPV positivity 2,34,35.

### Statistical analysis

Data were analysed by unpaired *t*-test, *P* < 0.05 were considered statistically significant. Results are presented as mean ± SD.

## Results

### Induction of heparanase expression in HNSCC cell lines by HPV16- derived E6

**Figure 1:**
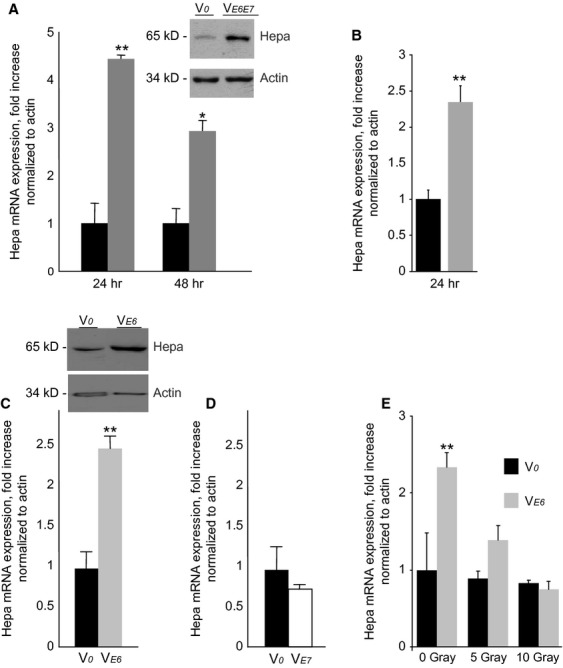
Effect of HPV16 oncogenes on heparanase expression in oral squamous cell carcinoma cells. (A and B) CAL-27 (A) and SCC-25 (B) cells were transiently transfected with the expression vector encoding for both HPV16 E6 and E7 oncogenes (V_*E6E7*_, grey bars), or with the corresponding control empty vector (Vo, black bars), as described in ‘Methods’. Twenty-four and 48 hrs later heparanase (Hepa) expression was assessed by qRT-PCR determination of mRNA levels and by immunoblotting (A, inset). (C) E6 oncogene is responsible for induction of heparanase in oral squamous carcinoma cells. CAL-27 cells were stably transfected with the expression vector encoding for HPV16 E6 (V_*E6*_, grey bars), or with the empty vector (Vo, black bars). Heparanase levels were measured by qRT-PCR (C) and immunoblotting (C, inset), as described in ‘Methods’, **P < 0.005. The results are representative of three independent experiments. (D) Transfection of CAL-27 cells with the expression vector encoding for HPV16 E7 (V_*E7*_, empty bars) does not affect heparanase levels, as compared with Vo-transfected cells (black bars). (E) Ionizing radiation inhibits heparanase expression in CAL-27 V_*E6*_, but not CAL-27-Vo, cells. Prior to irradiation, CAL-27-Vo (black bars) CAL-27 V_*E6*_ (grey bars) cells were maintained for 16 hrs in serum-free medium. The cells were then treated with the indicated doses of ionizing radiation. Six hours later, heparanase levels were measured by qRT-PCR. The experiment was repeated three times, and the results of one representative experiment done in duplicates are shown.

As HPV-driven tumourigenesis is primarily associated with viral oncoproteins E6 and E7, we initially investigated whether expression of E6/E7 genes in HPV-negative OSCC cell lines affects heparanase expression. For this purpose, human OSCC cells Cal-27 and SCC-25 were transfected with plasmid vector encoding for HPV16 oncoproteins E6 and E7 (V*e**6**e**7*) or with empty expressing vector pLXSN (*Vo*). Transfections were confirmed by RT-PCR analysis of E6 and E7 mRNA production using extracted cellular RNA (data not shown). Transiently transfected cells were then tested for heparanase expression at several time-points. As demonstrated in Figure 1A, transient expression of E6 and E7 resulted in significantly increased levels of heparanase mRNA and protein in Cal-27 cells. Similar results were obtained with additional HPV-negative OSCC cell line SCC-25 (Fig. 1B). Following selection, more than 100 stable-transfected clones were pooled (to avoid possible effects of insertional mutagenesis) and the cells were examined for heparanase expression. As shown in Figure S1, increased levels of heparanase mRNA and protein were detected in lysates prepared from Cal-27 cells stably transfected with V*e**6**e**7*, as compared to V*o-*transfected cells.

Next, to determine which of two HPV16 oncogenes is directly responsible for heparanase induction, we transfected Cal 27 cells with plasmid constructs encoding for either E6 (V*e**6*), E7 (V*e**7*) or with the empty vector (*Vo*). While transfection with E7 had no effect on heparanase expression (Fig. 1D), transfection with E6 resulted in a pronounced increase of both heparanase mRNA/protein levels in stable-transfected Cal-27 cells (Fig. 1C).

Given the ability of E6 to target p53, which acts as a powerful inhibitor of heparanase transcription 36, it is conceivable that in HPV-infected cells E6-mediated depletion of p53 relieves this inhibition, leading to overexpression of heparanase (Fig. S2). This assumption may explain, at least in part, improved response to chemo/radiotherapy in HPV-positive HNSCC patients 4: It is well-documented that DNA damage, imposed by ionizing radiation or cytotoxic drugs leads to increase in p53 levels. Thus, in HPV-positive tumours, radiation/chemo treatment may restore p53 content to the levels sufficient to inhibit heparanase expression and, consequently, heparanase-driven tumour progression. In support of this mode of action, treatment with clinically relevant doses of ionizing radiation significantly and dose-dependently decreased heparanase expression in Cal27-Ve6 but not in Cal27-Vo cells (Fig. 1E), while concomitant fourfold increase in the levels of p21/WAF1 gene (a well-characterized downstream effector of p53, data not shown) served as an indicator of increased p53 content.

### Co-localization of heparanase and p16 in human HNSCC specimens

**Figure 2:**
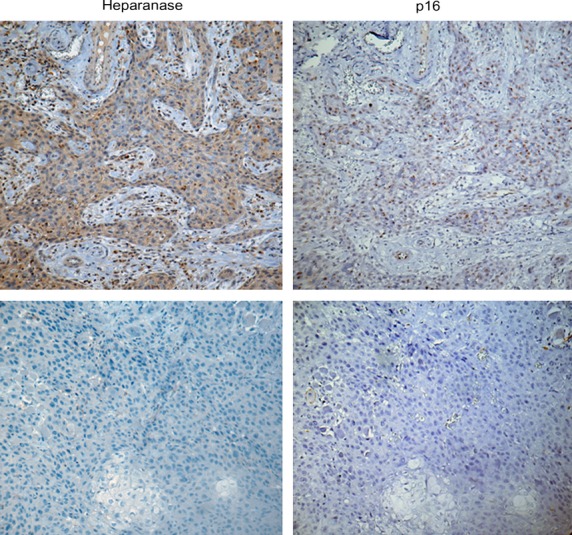
Coexpression of HPV infection marker p16 and heparanase in histological specimens of HNSCC tumours. Immunostaining with the antibodies specific for p16 (right panels) and heparanase (left panels) was performed as described in ‘Methods’. Note similarity in the spatial pattern of staining between heparanase and p16 (top panels), consistent with the involvement of HPV in heparanase induction. In contrast, 70% of p16-negative tumours were also heparanase negative (bottom panels). Magnification: ×200.

To validate the relevance of our *in vitro* findings in a clinical setting, we next examined spatial pattern of expression of p16 (a reliable surrogate marker for HPV infection 1) and heparanase in tissue specimens derived from HPV-positive OSCC. Among twelve p16-positive tumours examined, 10 (83.3%) were also positive for heparanase. Moreover, in all heparanase-positive tumours the pattern of p16 protein staining was similar to that of heparanase expression in defined areas of the tumour (Fig. 2, top panels). In contrast, in seven of 10 p16-negative tumours examined, no heparanase overexpression was detected (Fig. 2, bottom panels). Statistical analysis confirmed that p16-positive tumours are more likely to express heparanase (two-sided Fisher's exact test; *P* = 0.027). These findings, although limited by as small sample size, further support the role of HPV in induction of heparanase expression in HNSCC.

## Discussion

Despite favourable prognosis under treatment 1,2,4, HPV-positive HNSCC display aggressive phenotype, as exemplified by correlation between HPV positivity and tumour invasion depth/lymph node dissemination 3. Enzymatic degradation of ECM and, in particular, basement membranes, represents a universal mechanism of invasiveness and a pre-requisite for metastatic spread 37. Heparanase is strongly implicated in tumour invasion and metastasis, because of its ability to cleave HS, chief polysaccharide component responsible for maintaining barrier properties of the ECM and basement membranes 5–7,9,12. In light of the potential danger of inappropriate cleavage of HS in ECM, under physiological conditions the expression of the enzyme is kept tightly regulated and a majority of non-cancerous tissues are negative for heparanase 7,9,12, owing to constitutive inhibition of heparanase promoter by p53 36 and epigenetic modifications 38). In agreement with this notion, in head and neck tumours heparanase mRNA/protein are highly expressed, whereas normal epithelium expresses little or no heparanase, 23–27. Yet, the molecular pathways responsible for induction of heparanase expression in malignant tumours, *versus* lack of expression in normal tissues of the same origin (including HNSCC) 23,28,38 remain poorly understood. Transcription factors Sp1 and Ets were previously associated with basal activity of heparanase promoter 39–41, while early growth response 1 (EGR1) transcription factor 42 and oestrogen receptor 43 were implicated in inducible transcription of the heparanase gene in prostate and breast carcinomas (respectively). Our present data suggest that E6 oncogene is responsible for induction of heparanase in HPV-positive HNSCC. On the other hand, expression of the enzyme in three of ten HPV-negative tumours analysed in this study may be explained by alternative mechanisms involved in control of heparanase gene (*i.e*. inactivating mutations in p53 36). It is also conceivable that increase in heparanase level was not detected in a small fraction of HPV-positive tumours (two of twelve utilized in this study), again because of the action of alternative regulatory pathways (most likely—methylation of heparanase gene promoter 38).

Heparanase expression correlates with both invasiveness of HNSCC cell lines and the metastatic potential of head and neck tumours 23,24,26–28, emphasizing a role of the enzyme in HNSCC aggressive behaviour. This notion, together with our report here on induction of the heparanase gene by HPV16 E6, and the fact that HPV positivity also correlates with HNSCC aggressiveness 3, suggest that in HPV-driven oral tumourigenesis heparanase represents a novel downstream effector responsible for aggressive phenotype of HPV-positive HNSCC.

In summary, our results provide the first evidence for a functional involvement of HPV E6 in heparanase induction during head and neck tumourigenesis (most likely through p53-dependent mechanism) and may provide important avenue for future therapeutic exploration, relevant not only for HNSCC, but also for additional HPV-associated tumours, including cervical carcinoma (where heparanase overexpression is associated with aggressiveness and poor prognosis 44) and other types of lower genital tract neoplasms caused by HPV infection.

## Supporting information

Figure S1 Expression of heparanase in CAL-27 cells stably transfected with HPV16 E6 and E7 oncogenes.

Figure S2 Proposed mode of HPV E6-induced overexpression of heparanase.
