# Supplementary material for: Induction of heparanase by HPV E6 oncogene in head and neck squamous cell carcinoma
Source: J Cell Mol Med. 2013 Nov 28;18(1):181–6. doi: 10.1111/jcmm.12179 (PMC3916129; doi:10.1111/jcmm.12179)
Supplement: Figure S1 — Expression of heparanase in CAL-27 cells stably transfected with HPV16 E6 and E7 oncogenes. [file jcmm0018-0181-sd1.doc]

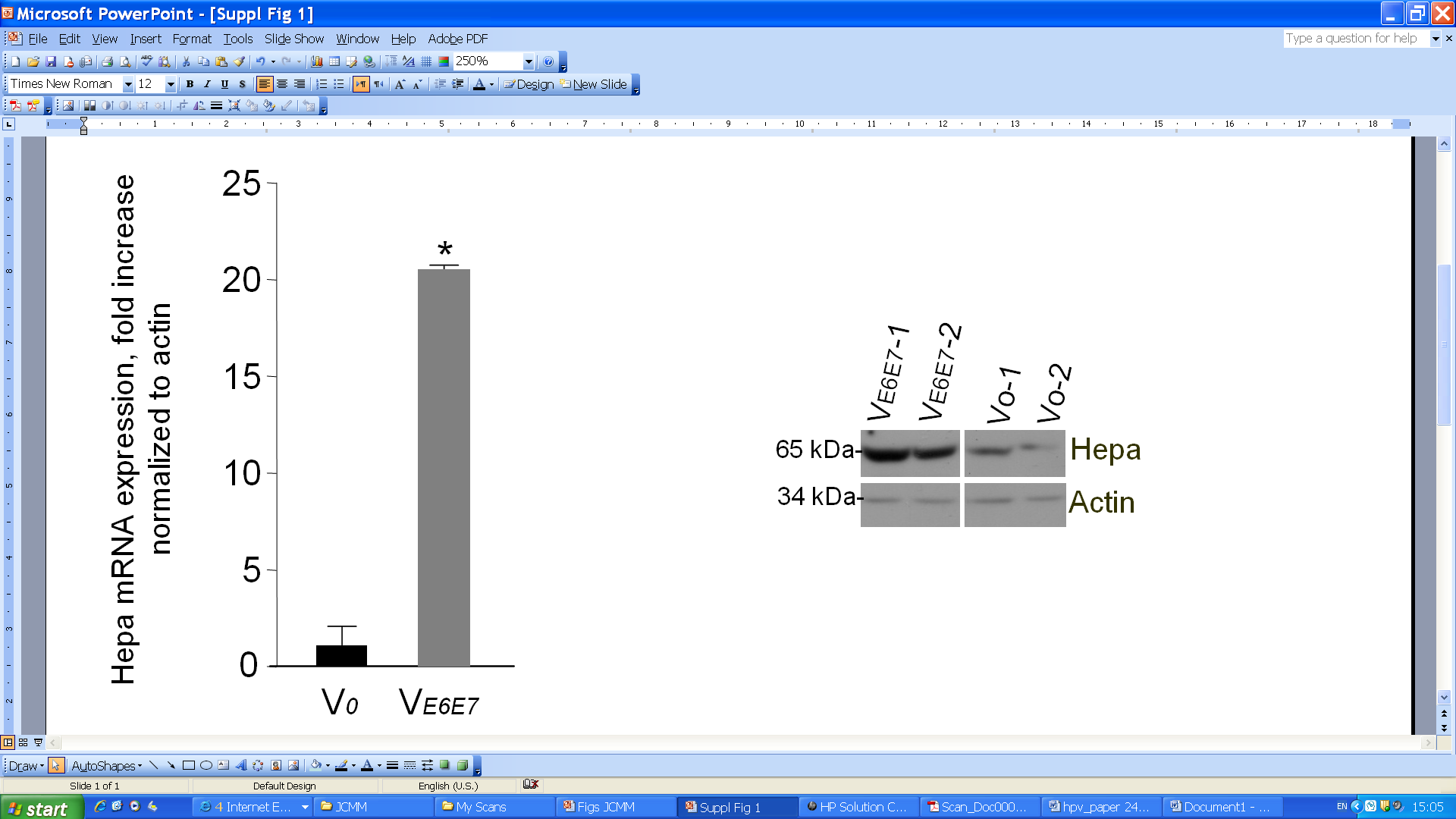


**Supplementary figure 1.** **Expression** **of heparanase in CAL-27 cells stably transfected with HPV16 E6 and E7 oncogenes**. Heparanase levels were assessed in several pools of clones of CAL-27 cells stably transfected with the expression vector encoding for both HPV16 E6 and E7 oncogenes (V*E6E7*, grey bars), or with the corresponding control empty vector (Vo, black bars), by qRT-PCR (left) and by immunoblotting (right). *p=0.013.
