# Supplementary material for: Induction of heparanase by HPV E6 oncogene in head and neck squamous cell carcinoma
Source: J Cell Mol Med. 2013 Nov 28;18(1):181–6. doi: 10.1111/jcmm.12179 (PMC3916129; doi:10.1111/jcmm.12179)
Supplement: Figure S2 — Proposed mode of HPV E6-induced overexpression of heparanase. [file jcmm0018-0181-sd2.doc]

**
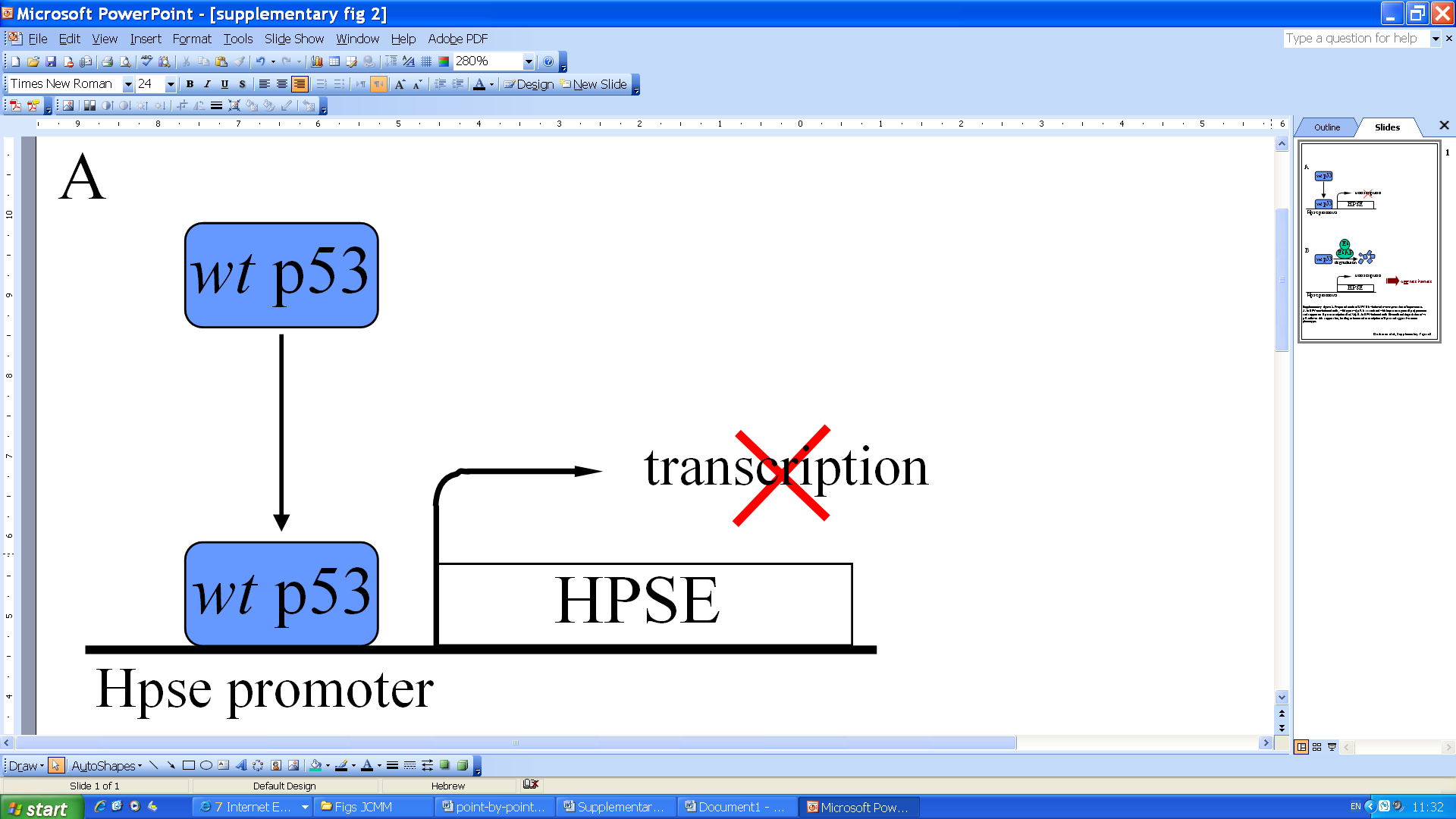

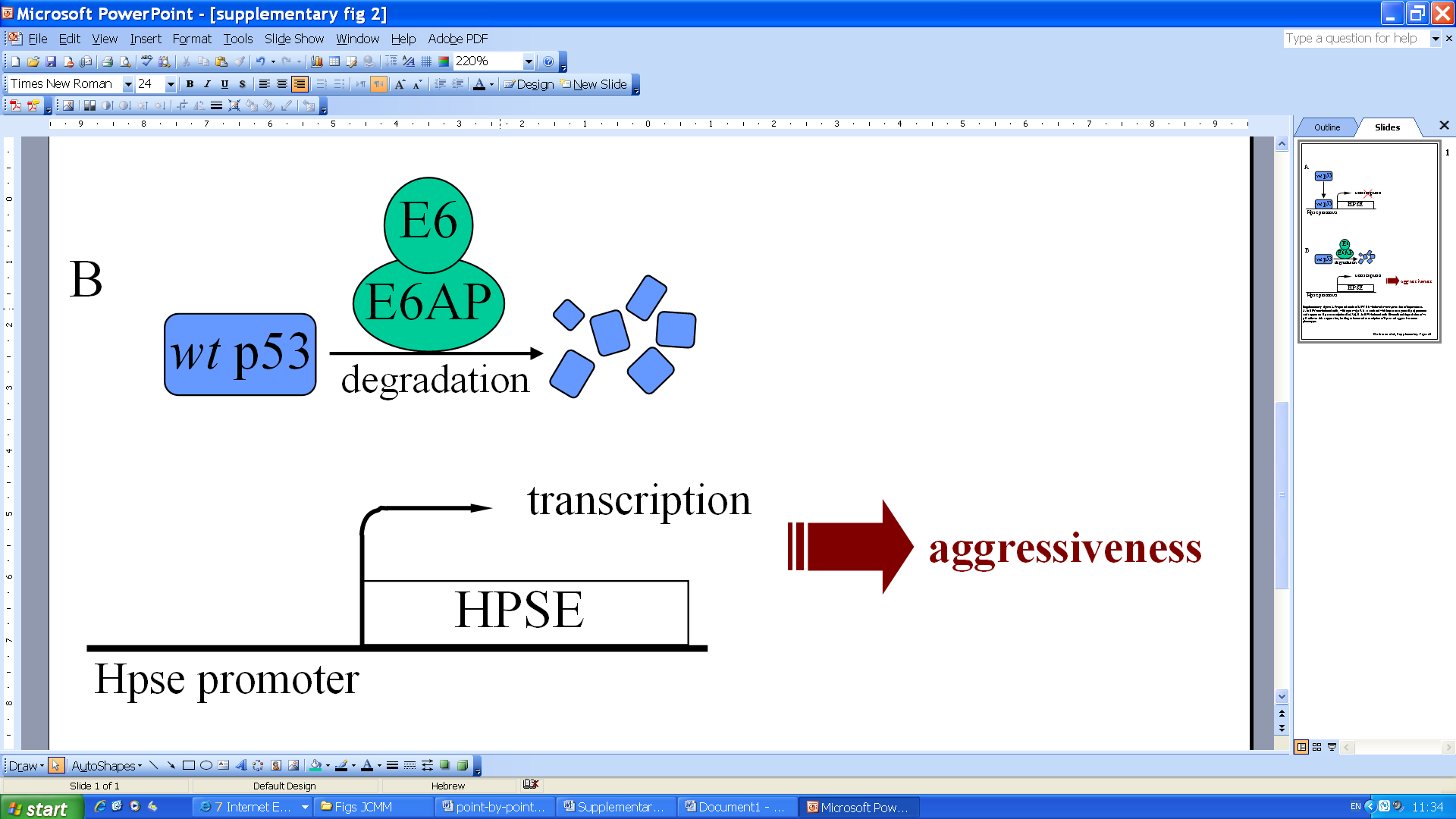
**

**Supplementary figure 2. Proposed mode of HPV E6 –induced overexpression of heparanase.**

**A.** In HPV-non-infected cells, wild type (wt) p53 is associated with heparanase gene (Hpse) promoter and suppresses Hpse transcription (Ref. 36). **B**. In HPV-infected cells E6-mediated degradation of *wt* p53 relieves this suppression, leading to increased transcription of Hpse and aggressive tumor phenotype.
